# Integrated approach for smart implantable cardioverter defibrillator (ICD) device with real time ECG monitoring: use of flexible sensors for localized arrhythmia sensing and stimulation

**DOI:** 10.3389/fphys.2013.00300

**Published:** 2013-10-24

**Authors:** Munish Puri, Kalyan C. Chapalamadugu, Aimon C. Miranda, Shyam Gelot, Wilfrido Moreno, Prashanth C. Adithya, Catherine Law, Srinivas M. Tipparaju

**Affiliations:** ^1^Department of Electrical Engineering, College of Engineering, University of South Florida, TampaFL, USA; ^2^Department of Pharmaceutical Sciences, University of South Florida College of Pharmacy, TampaFL, USA; ^3^Department of Pharmacotherapeutics and Clinical Research, University of South Florida College of Pharmacy, TampaFL, USA; ^4^Department of Cardiovascular Sciences, University of South Florida Morsani College of Medicine, TampaFL, USA

**Keywords:** ICD, defibrillator, arrhythmia, detection, flexible sensor array, heart

## Abstract

Arrhythmias are the most common cause of death associated with sudden death and are common in US and worldwide. Cardiac resynchronization therapy (CRT), evolving from pacemakers and development of implantable cardioverter defibrillator (ICD), has been adopted for therapeutic use and demonstrated benefits in patients over the years due to its design and intricate functionality. Recent research has been focused on significant design improvement and efforts are dedicated toward device size reduction, weight and functionality in commercially available ICD's since its invention in the 1960's. Commercially available CRT-D has shown advancement on both clinical and technical side. However, improved focus is required on the device miniaturization, technologically supported and integrated wireless based system for real time heart monitoring electrocardiogram (ECG). In the present report a concise overview for the state-of-the art technology in ICDs and avenues for future development are presented. A unique perspective is also included for ICD device miniaturization and integration of flexible sensing array. Sensor array integration along with its capabilities for identifying localized arrhythmia detection and targeted stimulation for enhancing ICD device capabilities is reviewed.

## Background

Since the advent of first artificial pacemaker, much has changed in the areas of electronics hardware, power efficiency, and programming algorithms (Hyman, 1932; Woollons, 1995). A recent survey forecasts that the cardiac implant market will grow by a single digit percent each year from 2010 through 2017, and reach estimated total revenue in excess of US $27 billion (Renub, 2013). The emergence of integrated circuit (ICs) in 1958 and Metal Oxide Semiconductor Field Effect Transistor (MOSFET) technology revolutionized the defibrillator product market. In recent years, Microsemi Corp. (Santa Ana, CA) developed the new high-voltage MOSFET for implantable cardioverter defibrillator (ICD), which is 40 percent smaller in size and functions as an overvoltage protection circuit in ICDs (Microsemi, 2001). American Heart Association reports that each month about 10,000 patients including children have ICD implanted to restore normal heart function (Dunbar et al., 2012). ICDs are lifesaving and generally produce satisfactory results, but reduce the quality of the life which has been primarily attributed to the technological limitations of the existing designs. Therefore, efforts to improve ICDs is essential toward producing smart next-generation devices that result in better patient satisfaction.

In the present report, we provide a concise overview of existing ICD technologies and discuss emerging design strategies and future improvements.

## Existing ICD technology

**Figure 1:**
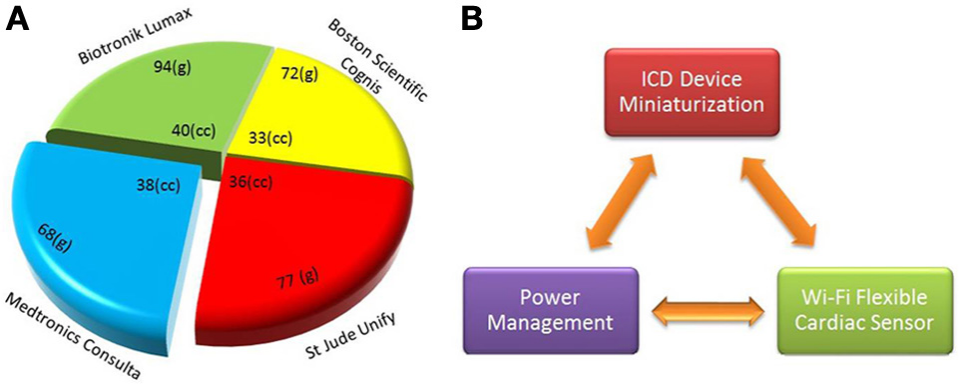
**Implantable cardioverter defibrillator (ICD) dimensional comparison and device integration scheme (A) size (cc) and weight (grams) of existing CRT-D devices by major market share (CRT-D, http://professional.sjm.com), (B) main components of proposed new generation ICDs are shown, such as device integration, power optimization, and Wi-Fi flexible sensor**.

The ICD is a device implanted inside the high risk patients to deliver the defibrillation pulse at the arrhythmia onset. The size and weight of commercially available cardiac resynchronization therapy-defibrillation (CRT-Ds) is shown in Figure 1A. Subcutaneously implanted ICD systems consist of a palm-sized metallic case and a set of leads. The metallic case includes two parts: pulse generator (electronics hardware like microprocessor) and a battery. The leads consist of silicone rubber with polyurethane outer protective coating to diagnose arrhythmia, i.e., detection of bradycardia and/or tachycardia.

## Limitations

In a report by Dunbar et al. (2012), concerns about patient's education and response toward ICD implants were noted. The report identifies that ICD implanted patients do not fully understand the ICD limitations related to battery, leads and electronics hardware.

### Battery

ICD devices are still large in size and shape (Sanders and Lee, 1996), often unable to meet the patient's demand for a miniaturized and smart devices. A major constraint to defibrillator size reduction is the larger battery dimensions. Current ICDs use lithium/silver-vanadium oxide batteries as a safe and clean energy source to power the implants, as per FDA class III medical device regulations (Crespi, 1993). In cathode-limited type of ICD batteries, automatic reforming of the capacitor takes 2 weeks of battery age to keep the low charge time (Skarstad, 2004).

### Leads

Leads convey information of cardiac rhythm to the ICD generator, and reciprocally deliver defibrillation therapy that is initiated by the pulse generator upon detection of the arrythmogenic events. Improper ICD lead design results in functioning defects (Lang et al., 1995). Lead malfunctioning causes inappropriate shocks, which may result in significant life threatening events and even death (Maisel and Kramer, 2008).

ICDs have become more technologically advanced since its invention in the 1960's. Despite the advancement and its effectiveness in terminating fatal arrhythmias, such as ventricular tachycardia (VT) and ventricular fibrillation (VF), these devices still have clinical limitations. The true incidence of device malfunction is not certain, but adverse events associated with ICDs can be obtained from FDA (Food and Drug Administration) reporting (MEDWATCH program) and observational studies. In one prospective study involving 778 patients with an ICD, common errors associated with the device were inappropriate shock therapy (16%) and ICD/lead dislodgement (4%) (Rosenqvist et al., 1998). Lead failure can also pose a problem resulting in the necessity for revision in 2.5% (95% CI 1.5–3.6) patients at the 5 years mark in one study involving 1317 patients (Eckstein et al., 2008). Lead malfunction can be due to a variety of causes such as wire fractures and insulation problems in the pace/sensing lead resulting in an inappropriate sensing or firing. When this occurs the pace/sensing lead must be replaced. However, if the malfunction is due to the high-voltage part of the lead, the entire defibrillator lead must be replaced. Investigators from this same study found that lead failure could be rectified by adding an additional pace/sense lead in 63% of the cases.

Previous reports also note that at times the ICD can produce a shock inadvertently for conditions other than VT or VF, often referred as inappropriate therapy. They may be burdensome to patients who receive painful shocks (Healey and Connolly, 2008; Mark et al., 2008). In the MADIT (Multicenter Automatic Defibrillator Implantation Trial) II inappropriate shocks due to wrong rhythm selection occurred in 11.5% of the patients with ICD's (Daubert et al., 2008). In these patients the trigger for inadvertent shocks mainly included atrial flutter/fibrillation, supraventricular tachycardia or abnormal sensing. The use of dual chamber devices can decrease the odds of inappropriate shocks vs. single chamber devices by sensing supraventricular arrhythmias (OR 0.53 95% CI 0.3–0.94; *P* = 0.03) (Friedman et al., 2006). Electrical noise, diaphragmatic potentials, and sensing of non-sustained VT are other examples when inappropriate therapy may occur (Germano et al., 2006).

Despite the proven benefits when an ICD is functioning properly, there is also evidence suggesting ICDs can cause structural damage, myocardial damage or even be arrythmogenic. In one small retrospective chart review, 41 patients who had severe tricuspid regurgitation due to ICD or permanent pacemakers (PPM) were evaluated (Lin et al., 2005). This study found that PPM and ICDs accounted for 17% of tricuspid valve leaflet perforations. Lead adherence to tricuspid valve and lead entanglement in the tricuspid valve occurred in 34 and 9% of patients, respectively. Evidence suggesting that ICDs have the potential to be proarrythmic is anecdotal. Hypothesis behind this theory is that the leads may cause local irritation of the myocardium. Additionally, due to poor sensing by ICD, inappropriate pacing can lead to ventricular arrhythmias. It is also well known that right ventricular pacing can exacerbate heart failure. In patients with that condition, this can increase the risk of ventricular arrhythmias (Germano et al., 2006). Twiddler's syndrome was first noted 45 years ago in some patients that developed a tendency to twist and turn the device (subconsciously or intentionally) in the surgical pocket, which can cause the leads to dislodge resulting in device failure. With smaller devices that can better fit the pocket space, this condition can be mitigated (Nicholson et al., 2003). Additional challenges include battery depletion, and electromagnetic interference from nearby wireless communication systems (Glikson and Friedman, 2001). Tissue growth on leads induces threats on signal sensing and leads displacement. Novel biomaterial coated sensing electrodes are a potential solution to prevent tissue growth and improved interfacing (Monteiro et al., 2009).

## Next generation integrated ICD

In order to improve the current ICD functionalities and size limitation, there is a need to follow a system of systems approach where hardware and software, such as analog (arrhythmia detection sensors) and digital system components, (microcontroller, RAM data storage) must be integrated as per overall system requirements. The ICD's central processing unit is a microprocessor which controls the output of the ICD unit depending on the information received from the system sensors.

### Device miniaturization

The main focus of ICD miniaturization is on comfortable fit device design and battery size reduction (Figure 1B). Important parameters in device miniaturization include battery volume, energy density and specific energy. Especially the latter two parameters are responsible for longer battery life in ICDs. To reduce the volume, the battery shape should conform to the internal ICD device geometry. Hybrid battery technology is progressing toward the use of lithium polymer materials, rechargeable and high-density thin-film batteries. Due to the advancements in nanotechnology, new generation microbatteries that occupy surprisingly less space are capable of delivering power and energy for longer periods of time (Xu et al., 2013). Charging through inductive coupling prolongs longevity, reduces space, and ultimately reduces the cost of surgery and chances of infection. Piezoelectric materials and ultrasonic energy could be used even under magnetic resonance imaging (MRI) environment (Karami and Inman, 2012).

### Flexible sensor array

Current ICD devices detect heart rhythm and sense arrhythmias using lead based system. The microprocessor's decision making capabilities depend on this arrhythmia information acquired from atrial and ventricular chambers. The major limitation of this approach is the lack of spatial resolution of information. For complete signal analysis and accurate decision making, the microprocessor requires information at the site of arrhythmia generation. This approach enhances the microprocessor's decision making skills and ICD's sensing capabilities as an integrated system.

The present state-of-the-art sensors are based on silicon complementary metal oxide semiconductor (CMOS) technology, which is rigid, non-conformal, and loses interface connections with tissue movement. Conformable and flexible sensors are able to interface soft, stretchable, and wet biological tissue. The flexible sensor technology, which is currently under research and development phase and being tested in animal models, would offer a promising solution in this area (Kim et al., 2012a). The flexible wireless sensors incorporated into the ICD device would be a step forward toward new generation ICDs (Sanders et al., 2005). The cardiac surface is curvilinear and soft, hindering the development of devices for bio-integration. The integrated array consists of flexible transistorized (FETs) sensors for measuring electrophysiological signals from the outer surface of the heart (Kim et al., 2012b). The programmed algorithm in the ICD detects and decides whether pacing or defibrillation is needed and accordingly the control signal is delivered. The sensor array can be advantageous for large surface area sensing, spatial resolution, and arrythmia localization. The impulses can be delivered by ICD leads or sensor array depending upon the origin of the arrhythmia. The cardiac sensing array can be designed with a separate power source to meet the minimum computing requirements of flexible sensors. The engineering design of the flexible sensor array may allow for an intact and conformal tissue-electronics interface. Battery power optimization needs to be studied to create flexible Wi-Fi cardiac sensor with optimal onsite memory (flexible RAM) options. Luther et al. demonstrated that applying a series of five small shocks, instead of single large one, reduces the energy amount needed by approximately 84% and reset the heart arrhythmias (Luther et al., 2011).

**Figure 2:**
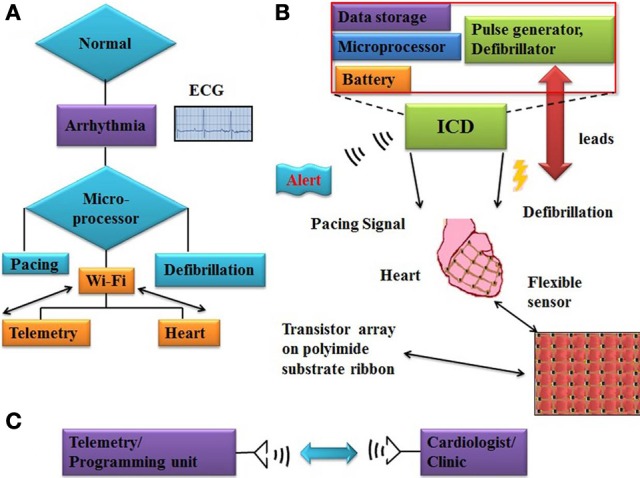
**Work flow chart of ICD functioning and flexible sensor array system (A) flow chart illustrates working principle of ICD and alert signal activation, microprocessor trigger pacing or defibrillation impulse on arrhythmias onset (B) major internal building blocks of ICD device, like battery, microprocessor, and RAM memory**. Flexible sensor array which consisting of transistors (FETs) fabricated on polyimide flexible substrate. **(C)** Block diagram of telemetry/programming unit to record clinically relevent data and connected with cardiologist/clinic for any emergency.

Electrocardiograms (ECG) detect heart abnormlities only at the time of the diagnostic test. Advanced micro processing chips are available for onsite sensing and decision making to detect various body movements, blood pressure, and pulmonary fluid. Figure 2 shows the complete ICD device miniaturization and wireless flexible sensor integration scheme. Wi-Fi based integrated sensors collect real-time data and transmit noise free original electrical signals from the cardiac surface. Multi-sensor array provides opportunities for understanding pulse pattern, local tissue behavior on defibrillation, diagnosing arrhythmias, and controlled tissue stimulation. Noise cancelling algorithms and circuits that can discriminate electromegnetic interference (EMI) must be designed to prevent the ICDs from triggering undesirable impulses. Supplying power through remote source using ultrasound is a novel concept under investigation for its application to ICD (Cheung and Neyzari, 1984). Piezoelectric materials based biasing systems can also be an alternative energy source (Ward et al., 2009).

### Wireless sensing data transfer

Current sensing and data transfer rely on leads, which is a major variable of concern in successfully ICD function. In order to circumvent these lead related limitations, wireless sensing array integration to ICD is a novel step toward collecting high resolution and quality signal information. Onsite computing (i.e., signal amplification) and ECG analysis are useful applications toward sensor design engineering which can be used for various biomedical applications. Real-time ECG monitoring and wireless programmability provide immediate information to healthcare providers to extend beyond the capabilities of conventional ICDs. Further development of advanced algorithms to detect and differentiate between fatal and non-fatal arrhythmia and to deliver proportionate shock will be an additional dimension toward inventing the new generation of smart ICDs. Telemetric synchronization of ICDs for the transmission of clinically useful data to doctors can be useful to handle any emergency situation.

In the present review an overview of device miniaturization, better sensing, impulse delivery, data transfer methods, and device integration information is provided for designing new generation smart device.

### Conflict of interest statement

The authors declare that the research was conducted in the absence of any commercial or financial relationships that could be construed as a potential conflict of interest.
